# Lipid Profile Altered in Phenanthrene Exposed Zebrafish Embryos with Implications for Neurological Development and Early Life Nutritional Status

**DOI:** 10.1021/envhealth.3c00002

**Published:** 2023-05-30

**Authors:** Victoria McGruer, Anil Bhatia, Jason T. Magnuson, Daniel Schlenk

**Affiliations:** † Department of Environmental Sciences, University of California, Riverside, California 92521-9800, United States; ‡ Metabolomics Core Facility, IIGB, University of California, Riverside, California 92521-9800, United States; § Department of Chemistry, Bioscience and Environmental Engineering, University of Stavanger, Stavanger 4021, Norway

**Keywords:** phenanthrene, lipid profiling, developmental toxicity, oil spills, toxicity mechanisms, *Danio rerio*

## Abstract

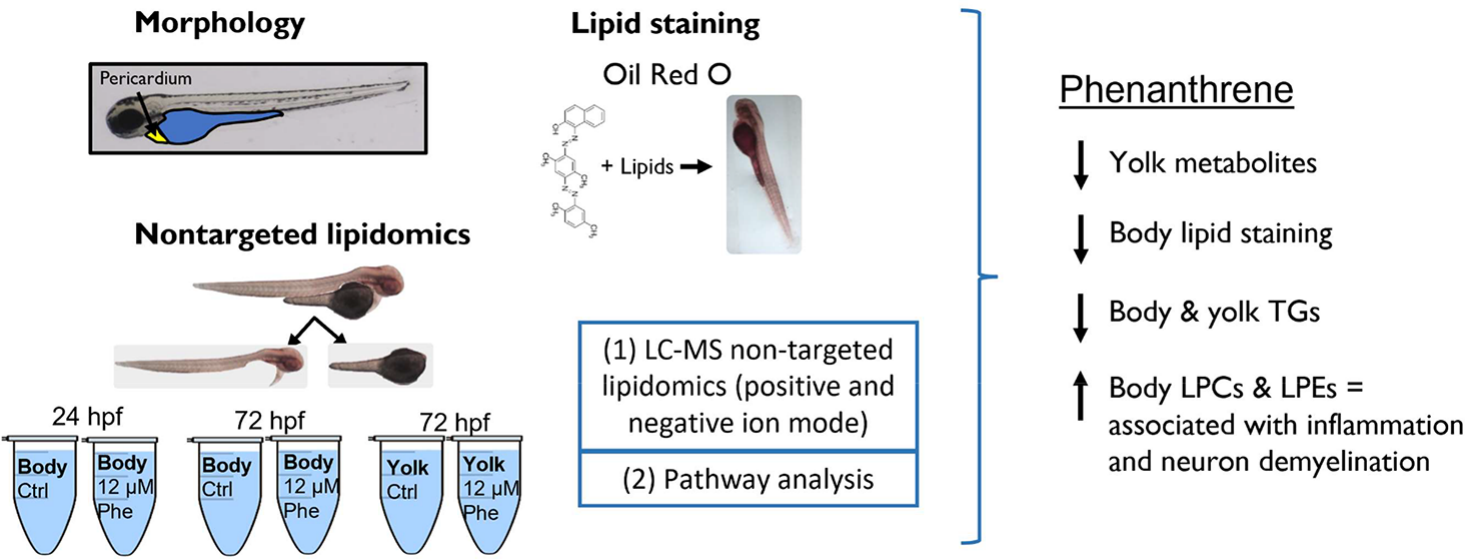

Lecithotrophic fish embryos rely on finite maternally deposited yolk resources for early development. Toxicant exposure can disrupt the uptake of yolk resources with consequences for development. In this study, we investigate the impacts of altered yolk utilization on fish embryos using the cardiotoxic compound phenanthrene. Zebrafish embryos were exposed to a cardiotoxic concentration of phenanthrene beginning at 6 hpf (hours post-fertilization) until a maximum of 72 hpf. Embryos were stained with Oil Red O to visualize neutral lipids. We then used a nontargeted approach to profile lipids in 24 and 72 hpf embryos after phenanthrene treatment. To assess changes in lipid movement within the embryo, the yolk sac was dissected from the body at 24 and 72 hpf and analyzed separately from the body at 72 hpf. Overall, total metabolites were significantly reduced in the yolk sac, and staining for neutral lipids was reduced in the embryo body at 72 hpf. This result is consistent with significant reductions in triglycerides in both the embryo body and yolk, indicating a limited contribution of impaired cardiac function to lipid mobilization at the dose tested. Additionally, lysophosphatidylcholines and lysophosphatidylethanolamines were significantly increased in the 72 hpf embryo body. Bioinformatic pathway analysis indicated that changes to these lysophospholipids could be linked to a disease model associated with inflammation and neuron demyelination consistent with previously observed injuries to neuronal and eye development in fish embryos and larvae.

## 1 Introduction

Developing organisms are uniquely vulnerable to toxicant exposure, and disruption of biological function at critical early life stages can cause permanent defects that persist to adulthood. Lecithotrophic fish embryos are wholly reliant on the proteins and lipids supplied by the maternally deposited yolk sac, as this is the only source of energy and nutrition until the fish has sufficiently developed to feed exogenously. Lipids have essential functional roles in early development as energy stores, for membrane formation and function, and in signaling processes. Therefore, disruption of nutrient transfer and the lipid profile during embryo development could underlie other developmental abnormalities.

Decades of research have shown that early life-stage fish, across many species, are susceptible to crude oil and the polycyclic aromatic hydrocarbons (PAHs) found in oil.^1^ Phenanthrene, a model tricyclic PAH found in crude oil, produces an indistinguishable phenotype in developing fish from whole oil, and mixture studies have shown relative toxicity increases with the percentage of tricyclic compounds.^2^ phenanthrene is listed as a priority pollutant by the US Environmental Protection Agency and is commonly detected in the aquatic environment and urban air.^3^ Studies have reported water concentrations up to 3.32–6.61 μg/L in the Western Bengal basin in India^4^ and from 0.019 μg/L to 2.05 μg/L in the Bohai region, China.^5^ Phenanthrene or oil-exposed fish embryos display pericardial edema, reduced heart function, and craniofacial defects.^2^ Other impacts include altered eye development^6−8^ and various behavioral end points,^9,10^ which may be caused by altered neurological development and function.^11^ Understanding the mechanisms underlying these phenotypes will allow better predictions regarding interspecies sensitivity and associated apical end points used in ecological risk assessments such as survival, growth, and reproduction.

Studies have shown that crude oil, or phenanthrene alone, can rapidly impair the function of isolated fish cardiomyocytes by disrupting ion flux.^12,13^ Follow-up in vitro mutagenesis experiments demonstrated that this is likely due to specific interactions with the cardiac Ether-a-go-go-Related Gene (ERG) encoded K^+^ channels.^14^ However, early life-stage exposure to phenanthrene or crude oil impacts numerous organs in addition to the heart. The enduring question is whether some or all of these outcomes are downstream of specific cardiac defects.

To investigate possible mechanisms and toxicity pathways, numerous studies have assessed the impact of oil and phenanthrene on the transcriptome in developing fish embryos. Several of these studies highlight cholesterol and lipid metabolism as top impacted pathways.^8,15−17^ A few studies have investigated the impacts of crude oil on the lipidome specifically. Sørhus et al. (2021)^8^ profiled lipids in early life stage Atlantic haddock and focused on fatty acid distribution. Additionally, Laurel et al. (2019)^18^ exposed polar cod (*Boreogadus saida*) embryos to low concentrations of crude oil for 3 days and saw disruption to triacylglycerides (TG), free fatty acids, sterols, and total lipids at hatching stage when embryos are still dependent on a maternally deposited yolk sac. Remarkably, TGs were still altered after the transition to exogenous feeding until the last stage tested (approximately 170 days post-exposure). Together, this demonstrates that the effects of oil on lipid homeostasis are persistent and may underlie enduring impacts of exposure on growth and survival at later life stages.

One hypothesis for lipid disruption in fish during early development is that the embryo cannot mobilize nutrients from the yolk due to oil-induced cardiac disruption. Both yolk sac stage Atlantic haddock (*Melanogrammus aeglefinus*)^16^ and zebrafish (*Danio rerio*) embryos^19^ display a yolk sac that is larger than controls following exposure to crude oil and phenanthrene, respectively. The present study looks to test the hypothesis that underutilization of the yolk drives changes to the lipid profile in yolk sac stage fish. Furthermore, we aim to understand how altered lipid homeostasis impacts the nutritional status of early life-stage fish and how developmental and signaling processes may be altered during organogenesis.

## 2 Materials and Methods

### 2.1 Animals

Adult wild-type zebrafish were maintained and bred per Institutional Animal Care and Use Committee-approved animal use protocol (No. 20190003) at the University of California, Riverside. The water temperature of breeder tanks was monitored daily and maintained at 27–28 °C. Ammonia, nitrate, nitrite, hardness, and pH were monitored weekly with test strips. Zebrafish were fed daily with a dry diet (Gemma Micro 300, Skretting, Fontaine-lès-Vervins, France). Fertilized eggs were collected within 15 min of spawning and raised in a temperature-controlled incubator. Adult tanks and embryo dishes were maintained under a 14:10 h light–dark cycle. All embryos were sorted and staged according to Kimmel et al. (1995).^20^

### 2.2 Embryo Exposures

Embryo exposures followed the protocol described in McGruer et al., 2021.^19^ In brief, 12 μM phenanthrene and 0.06% DMSO solvent control exposure solutions were prepared in embryo water from stock solutions immediately before exposure. Shield-stage (6 hours post-fertilization (hpf)) zebrafish embryos were then randomly distributed with forceps into a 48-well polystyrene plate (Corning) so that each well contained one embryo with 0.667 mL of exposure solution. Exposures were renewed with 0.5 mL of freshly prepared solution at 24 and 48 hpf, and embryos were sampled as late as 72 hpf. Sampling targeted stages of developmental known to be sensitive to phenanthrene exposure^2^ at concentrations that produce cardiac deformities by 72 hpf.^19^ Embryos were pooled into replicate samples for lipidomics analyses or individually stained and/or imaged for morphological and Oil Red O analysis. McGruer et al. (2021)^19^ used the same exposure methods and tested multiple doses. Phenanthrene water concentrations are quantified and reported in that paper.

### 2.3 Morphological Assessment

Seventy-two hours post-fertilization embryos were arranged laterally in 4% methylcellulose and imaged using Leica MZ10F stereoscope equipped with a DMC2900 camera. Images were imported into ImageJ (Ver 1.52p; National Institutes of Health), and the pericardial area was quantified.

### 2.4 Sample Collection for Lipidomics Analysis

Nontargeted lipidomics was used to obtain an unbiased snapshot of the embryo body separate from the unused yolk sac. At 24 hpf, the time point when a rudimentary heartbeat can be detected in the embryo, the yolk sac was removed to profile lipids in the embryo body alone. At 72 hpf, when phenanthrene-induced cardiac disruption is pronounced, lipids were profiled in both the embryo body and the remaining yolk sac separately to draw conclusions about the embryo’s capacity to transfer nutrients from the yolk to the developing fish body.

First, 24 hpf embryos were dechorionated using 1 mg/mL Pronase before sample collection. Twenty-four or 72 hpf embryos were collected in a 1.5 mL microcentrifuge tube and placed on ice for 2 min for anesthetization. After anesthetization, the remaining embryo water was removed, and embryos were washed with deyolking buffer (55 mM NaCl, 3.6 mM KCl, 1.25 mM NaHCO_3_)^22^ to remove any remaining chorion particles and then placed on ice for another 5 min. Seventy-two hpf embryos were then pipetted through a 200 μL pipette tip to disrupt and dissociate the yolk. The 24 hpf embryos were more delicate and pipetted a few times through a 1 mL pipette tip until the yolk was visibly dissociated. The samples were transferred to a glass plate. Under a stereoscope, the embryo bodies were confirmed to be intact, and any excess remaining yolk was removed using a scalpel before collection. Yolk samples were collected for the 72 hpf time point by removing all embryo bodies from the glass plate before collecting the remaining solution containing the yolk using a glass pipette. The glass plate was then washed with an additional 250 μL of deyolking buffer, which was added to the sample to maximize recovery. All samples consisted of 60 pooled embryos (body or yolk) per technical replicate. Five technical replicates were analyzed per exposure group. Due to difficulty removing the yolk from 24 hpf embryos, three batches of 20 embryos were collected per replicate to prevent degradation during sample collection. Each sample was visually inspected to ensure the yolk was completely separated from the embryo body.

### 2.5 Liquid Chromatograpy Mass Spectrometry (LC-MS) Lipidomics

A biphasic approach was used to extract lipids from the samples.^23^ In summary, the samples were freeze-dried and 100 μL/mg of ice-cold 3:2 methyl *tert*-butyl ether/80% methanol was added to the sample in a glass vial. The samples were then sonicated for 20 min in a cold-water bath and vortexed for 90 min at 4 °C. Next, 200 μg/mL of LC-MS grade water was added to cause phase separation, followed by a 5 min vortex at 4 °C. The samples were then centrifuged for 15 min (4000 xg, 4 °C). The top 200 μL of extract, the nonpolar layer, was transferred to a glass vial and dried under nitrogen at room temperature. The nonpolar fraction was resuspended in 200 μL of 9:1 methanol/toluene for analysis. Finally, 20 μL was pooled from each sample to prepare a quality control sample.

LC-MS lipidomics analysis was performed at the UC Riverside Metabolomics Core Facility as described previously,^24^ with minor modifications. Briefly, analysis was performed on a Waters G2-XS quadrupole time-of-flight mass spectrometer coupled to a Waters Acquity I-class UPLC system. Separations were carried out on a Waters CSH C18 column (2.1 × 100 mm, 1.7 μM). The mobile phases were (A) 60:40 acetonitrile/water with 10 mM ammonium formate and 0.1% formic acid and (B) 90:10 isopropyl alcohol/acetonitrile with 10 mM ammonium formate and 0.1% formic acid. The flow rate was 400 μL/min, and the column was held at 65 °C. The injection volume was 1 μL. The gradient was as follows: 0 min, 10% B; 1 min, 10% B; 3 min, 20% B; 5 min, 40% B; 16 min, 80% B; 18 min, 99% B; 20 min 99% B; 20.5 min, 10% B. The MS scan range was (50 to 1600 *m*/*z*) with a 100 ms scan time. MS/MS was acquired in a data-dependent fashion. The source and desolvation temperatures were 150 and 600 °C, respectively. Desolvation gas was set to 1100 L/h and cone gas to 150 L/h. All gases were nitrogen except the collision gas, which was argon. The capillary voltage was 1 kV in positive ion mode and 2 kV in negative ion mode. The quality control sample was analyzed periodically to monitor system stability and performance. Samples were analyzed in random order. Leucine enkephalin was infused and used for mass correction.

### 2.6 Lipidomics Data Processing

The LC-MS raw spectral data files were converted to analysis base file (Abf) format using ABF Converter and then were imported into MS-DIAL.^25^ The postprocessing processes (peak picking, alignment, deconvolution, integration, and spectral matching) were performed in MS-Dial. Ions included for deconvolution were [M + H]^+^, [M + NH_4_]^+^, [M + Na]^+^, [M + K]^+^, and [M + H – H_2_O] in positive ion mode and [M – H], [M + H_2_O]^−^, [M – 2H]^2–^, and [M + HCOO]^−^ in negative ion mode. The untargeted data were normalized to the total ion count. Features with a cyclic voltammetry greater than 30% across QC injections were removed from the analysis.^26,27^ Mass Bank of North America^28^ and Lipidblast^29^ databases were used for lipid annotations.

Data sets from positive and negative ion mode analysis were combined. If a metabolite was identified in both ion modes, the mode demonstrating the higher abundance of the metabolite in the sum of the quality control samples was kept for analysis. Known metabolites were matched with HMDB and PubChem CID identifiers using lipid maps and the Human Metabolome Database.

### 2.7 Oil Red O Staining

For Oil Red O staining, embryos were sampled at 24, 27, 30, 48, and 72 hpf. Multiple stages between 24 and 72 hpf were selected to investigate how neutral lipid abundance changes throughout early development. Embryos were dechorionated if necessary and then fixed overnight at 4 °C with freshly prepared 4% paraformaldehyde/1× phosphate-buffered saline (PBS). Following fixation, samples were washed three times each with 1× PBS and then stored in 1× PBS at 4 °C until staining. Oil Red O staining followed methods described by Reddam et al., 2019. In brief, fixed embryos were soaked in increasing concentrations of propylene glycol and then incubated overnight in 0.5% Oil Red O in propylene glycol. The following day, stained embryos were washed with decreasing concentrations of propylene glycol, transferred to 1× PBS, and stored at 4 °C until imaging. Stained embryos were arranged laterally in agarose molds and imaged. Analysis of still frames was conducted in ImageJ. Images were color-inverted, and the mean stain intensity was quantified in the embryo head and trunk.

### 2.8 Statistical and Pathway Analyses

Differences in morphological, staining, and lipid class data were statistically tested in R.^30^ Changes to the pericardial area between treatment groups were determined by the nonparametric Wilcoxon rank sum test. Mean 95% confidence intervals for Oil Red O staining and lipid class data were determined through bootstrapping (resampling with replacement; *n* = 10,000). Significant differences were determined based on overlap in confidence intervals.

Metabolomics data was imported into MetaboAnalyst version 5.0 (https://www.metaboanalyst.ca/).^31^ The data were log-transformed (base 10), and no further filtering was applied to the data set. MetaboAnalyst was further used to determine differentially expressed metabolites, calculate fold-change, and produce principal component analysis (PCA) score plots. Significantly different metabolites (unadjusted *p* value) were uploaded to Ingenuity Pathway Analysis (Qiagen) for pathway enrichment analysis. All in-text data are expressed as the mean ± standard deviation.

## 3 Results

### 3.1 Effects of Phenanthrene on Survival and Pericardial Area

**Figure 1:**
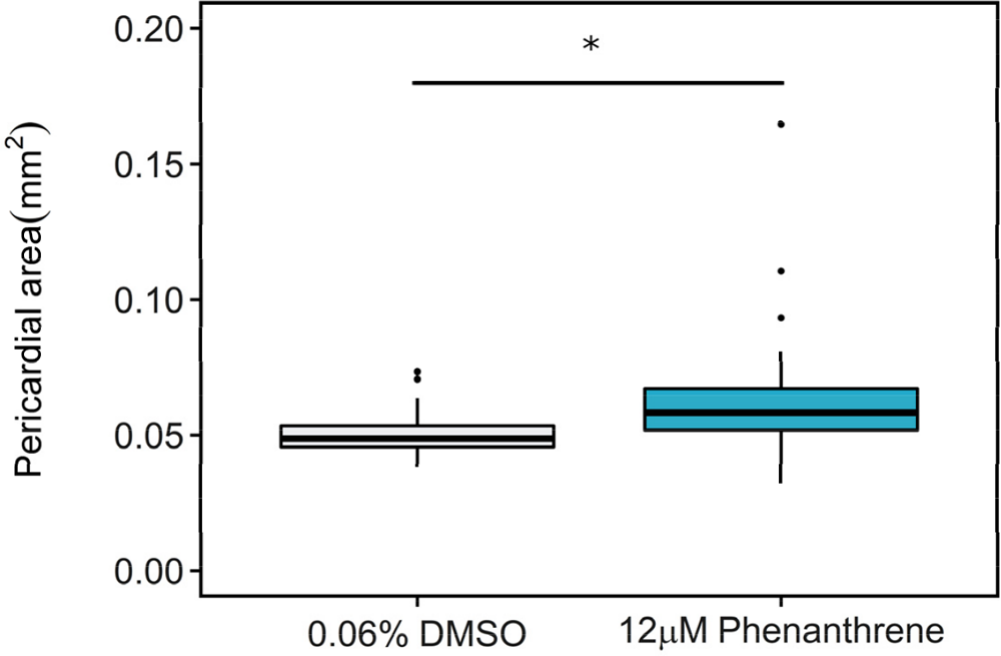
Pericardial area (mm^2^) in 72 hpf zebrafish embryos exposed to 0.06% DMSO control (*n* = 32) or 12 μM phenanthrene (*n* = 28). Data were analyzed by Wilcoxon rank sum test; *p < 0.05.

No mortality was observed by 24 hpf with 12 μM phenanthrene treatment. Percent survival at 72 hpf was 99.3 ± 1.0% in the DMSO control group and 94.6 ± 7.2% in the 12 μM phenanthrene treatment (Figure S1). The pericardial area was measured to assess the development of phenanthrene-induced cardiotoxicity. Exposure to 12 μM phenanthrene significantly increased the pericardial area in embryos (0.06 ± 0.03 mm^2^) at 72 hpf relative to the 0.06% DMSO control (0.05 ± 0.01 mm^2^) (*p* = 0.012) (Figure 1).

### 3.2 Time Course for Oil Red O Staining

**Figure 2:**
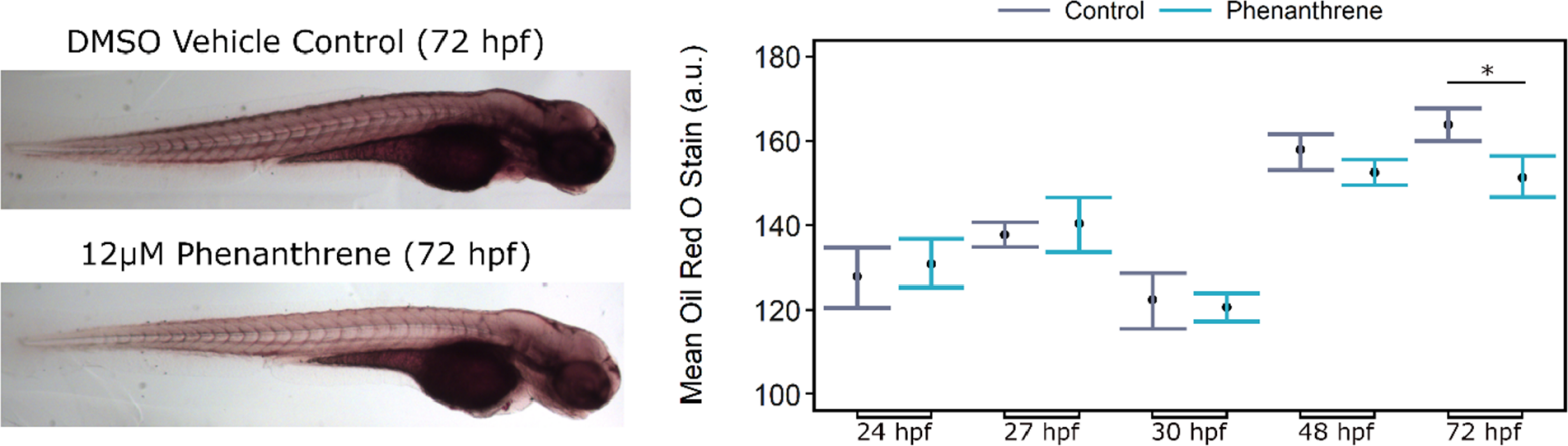
(A) Representative images of 72 hpf zebrafish embryos stained with Oil Red O. (B) Mean Oil Red O staining measured in the body of the embryo (head and trunk) excluding the yolk at 24, 27, 30, 48, and 72 hpf (*n* = 8–23). Errors bars represent the bootstrapped 95% confidence intervals surrounding the mean; *p < 0.05.

Oil Red O staining was used to visualize neutral lipids in the embryo body across several time points from 24 to 72 hpf. Mean staining intensity was not significantly affected between the control and phenanthrene-exposed groups at 24, 27, 30, or 48 hpf. However, by 72 hpf, there was a significant reduction in staining in the 12 μM phenanthrene exposed embryos relative to the DMSO control (*p* < 0.05) (Figure 2B). Representative images are provided in Figure 2A.

### 3.3 Nontargeted Lipidomics—Known and Unknown Metabolites

**Figure 3:**
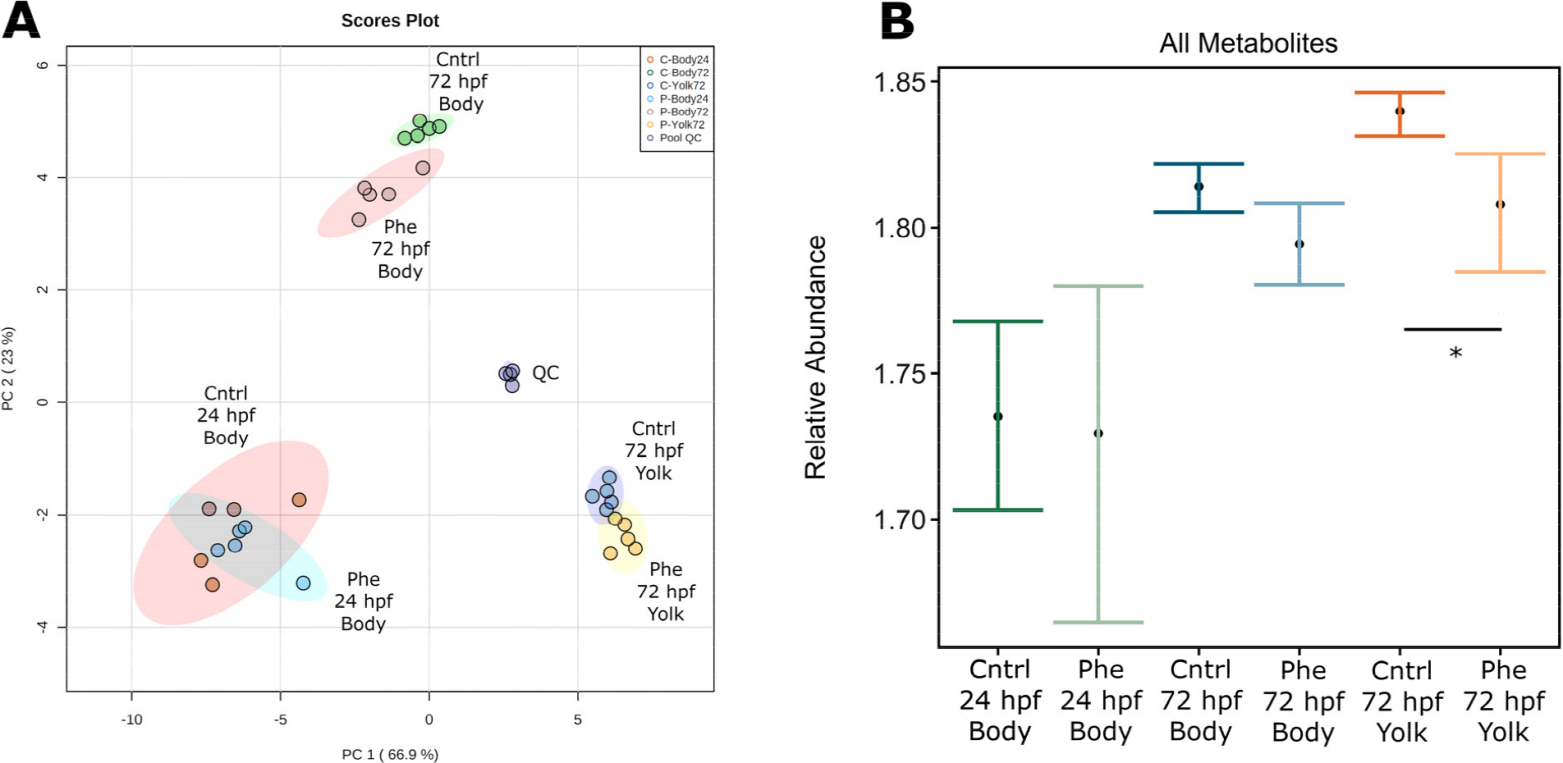
(A) Principal component analysis (PCA) considering all metabolites, both known and unknown. Highlighted areas represent 95% confidence regions. (B) Total metabolites, both known and unknown, within each treatment and time point. Errors bars represent the bootstrapped 95% confidence intervals surrounding the mean. Each group contains *n* = 5; *p < 0.05.

All known and unknown metabolites were used for principal component analysis. The first component axis explained 66.9% of the variation, and the second axis explained 23%. The body samples from 72 hpf embryos exposed to the vehicle control or 12 μM phenanthrene exposure solutions are significantly separated. The 72 hpf yolk samples are mostly separated by exposure, though the 95% confidence regions overlap. The 24 hpf body samples are not separated by exposure (Figure 3A). Both known and unknown metabolites were summed to determine the total metabolite abundance within each sample. We found a significant reduction in total metabolite abundance in the yolk samples of 72 hpf phenanthrene exposed embryos relative to control embryos. There was a reduction in total metabolite abundance, though the 95% confidence intervals overlapped, in the 72 hpf body samples exposed to phenanthrene relative to the control (Figure 3B). Generally, metabolites that were low in abundance in the 72 hpf yolk were high abundance in the 72 hpf body and vice versa (Figure S2).

### 3.4 Nontargeted Lipidomics—Changes to Lipid Classes

**Figure 4:**
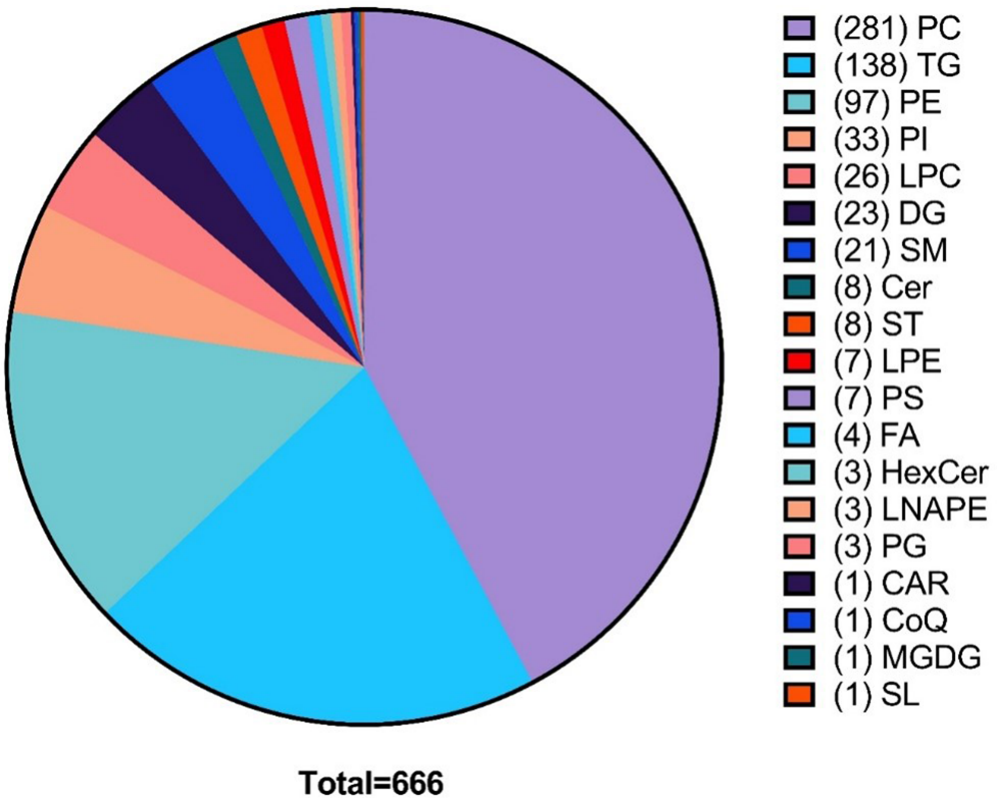
Number of lipids identified in each lipid class: phosphatidylcholine (PC), triacylglycerides (TG), phosphatidylethanolamine (PE), phosphatidylinositol (PI), lysophosphatidylcholine (LPC), diacylglyceride (DG), sphingomyelin (SM), ceramide (Cer), sterol (ST), lysophosphatidylethanolamine (LPE), phosphatidylserine (PS), fatty acid (FA), hexosylceramide (HexCer), *N*-acyl phosphatidylethanolamine (LNPE), phosphatidylglycerol (PG), acyl carnitine (CAR), coenzyme Q (CoQ), monogalactosyldiacylgylcerol (MGDG), saccharolipids (SL).

The nontargeted analysis revealed 7563 metabolites, of which 666 could be identified. Figure 4 displays the relative abundance and the total number of lipids identified within each class. Phosphatidylcholines (PCs) and triglycerides (TGs) comprised over half of the identified lipids. Of the identified metabolites, only 10 were significantly altered in the 24 hpf embryo body samples. However, by 72 hpf, 188 and 112 known metabolites were significantly altered following phenanthrene exposure in the embryo body and yolk, respectively (Table 1).

**Table 1:** Number and Direction of Significantly Altered Metabolites Comparing 12 μM Phenanthrene Exposure to Control Treatment within Each Sample Type

sample type	altered metabolites (*p* < 0.05)	direction of change (↑/↓)
24 hpf - body	10	2/8
72 hpf - body	188	58/130
72 hpf - yolk	112	35/77

**Figure 5:**
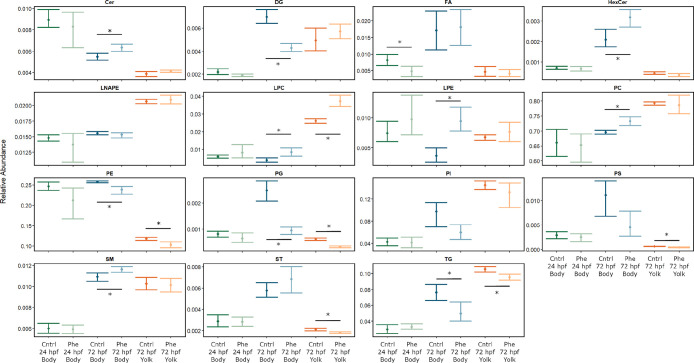
Relative abundance of metabolites by lipid class. Errors bars represent the bootstrapped 95% confidence intervals surrounding the mean. Data are grouped by treatment, developmental stage, and sample type. Each group contains *n* = 5: Ceramide (Cer), diacylglyceride (DG), fatty acid (FA), hexosylceramide (HexCer), *N*-acyl phosphatidylethanolamine (LNPE), lysophosphatidylcholine (LPC), lysophosphatidylethanolamine (LPE), phosphatidylcholine (PC), phosphatidylethanolamine (PE), phosphatidylglycerol (PG), phosphatidylinositol (PI), phosphatidylserine (PS), sphingomyelin (SM), sterol (ST), triacylglyceride (TG); *p < 0.05.

Next, we compared changes in overall metabolite classes between 72 hpf body and yolk samples to investigate how distinct classes of metabolites may be utilized in each of these compartments. Lysophosphatidylcholines (LPCs) were significantly increased with exposure in both the body and the yolk relative to the control fish. Phosphatidylethanolamines (PEs), phosphatidylglycerols (PGs), and TGs were significantly reduced with phenanthrene exposure in both the embryo body and yolk compartments. Ceramides (Cers), hexosylceramides (HexCers), lysophosphatidylethanolamines (LPEs), PCs, and sphingomyelins (SMs) were significantly increased in the embryo body of phenanthrene-exposed fish but were not altered in the yolk. Diacylglycerides (DGs) were significantly reduced in the body of phenanthrene-exposed fish relative to the control but were not altered in the corresponding yolk samples. Phosphatidylserines (PSs) and sterols (STs) were significantly reduced in the embryo yolk but not in the embryo body. Notably, no metabolite classes were significantly increased in the yolk without also being increased in the embryo body (Figure 5).

### 3.5 Pathway Analysis

**Figure 6:**
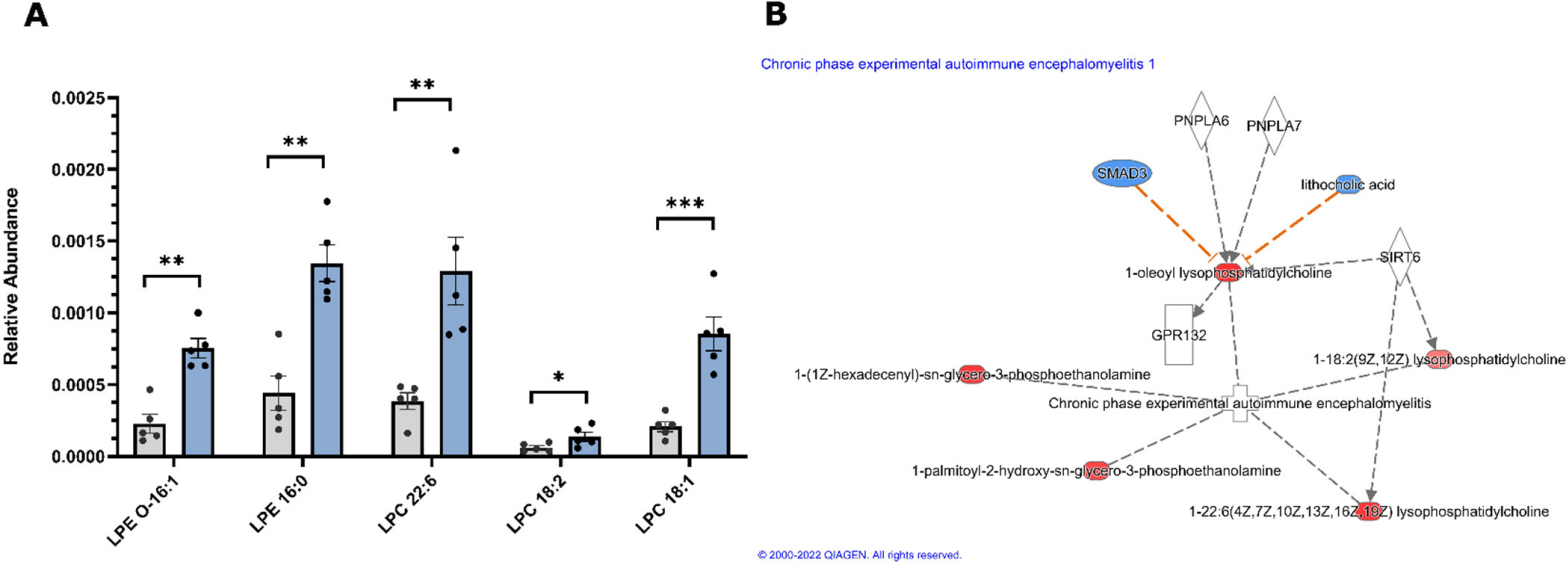
(A) Relative abundance of LPC and LPE species highlighted by Ingenuity Pathway Analysis in the 72 hpf zebrafish embryo body samples. Gray bars represent the DMSO control, and blue bars represent the 12 μM phenanthrene exposures. Points represent individual replicates (*n* = 5). Error bars represent mean ± SEM; *p < 0.05; **p < 0.01. (B) Lipids linked to the development of the disease model chronic phase experimental autoimmune encephalomyelitis by Ingenuity Pathway Analysis. Red indicates increased abundance, and white indicates predicted upstream regulators, or downstream events. The orange line predicts activation.

The top diseases and disorders predicted by IPA were immunological disease, inflammatory disease, inflammatory response, neurological disease, and organismal injury and abnormalities (*p* < 0.05) (Figure S3). These predictions were all associated with the development of experimental autoimmune encephalomyelitis (EAE), a commonly used experimental model for the human inflammatory neuronal demyelinating disease, multiple sclerosis (MS) (Figure 6). These findings were primarily driven by a significant increase in the abundance of several LPC and LPE species which were among the top impacted lipid species in the study (Table 2).

**Table 2:** Top Impacted Lipids in 72 hpf Embryo Body Samples^a^

metabolite name	FC	log2.FC.	raw.pval
DG 40:6|DG 18:0_22:6	0.44159	–1.1792	3.66 × 10^–5^
LPC 16:0	2.4703	1.3047	0.000703
LPC 18:1	4.1114	2.0396	0.000218
LPC 18:1 isomer3	2.7648	1.4671	0.048237
LPC 18:2 isomer2	2.244	1.1661	0.041288
LPC 20:5	3.4841	1.8008	0.003315
LPC 22:5 isomer1	2.5944	1.3754	0.034743
LPC 22:6	3.3451	1.7421	0.001832
LPE 16:0	3.0436	1.6058	0.00234
LPE 18:0	2.4579	1.2975	0.004025
LPE 22:6 isomer_2	3.0477	1.6077	0.002241
LPE O-16:1	3.3085	1.7262	0.001148
MGDG 32:0|MGDG 16:0_16:0	0.34448	–1.5375	0.000231
PG 34:1|PG 16:0_18:1	0.44159	–1.1792	0.000378
PG 36:2|PG 16:0_20:2	0.40394	–1.3078	0.001385
PG 44:12|PG 22:6_22:6	0.29452	–1.7635	0.00012
PI 40:6|PI 18:0_22:6	0.44514	–1.1677	0.026335
PI 40:7|PI 18:1_22:6	0.36494	–1.4543	0.000492
TG 58:11|TG 16:0_20:5_22:6	0.4989	–1.0032	0.024986
TG 59:12|TG 15:0_22:6_22:6	0.43362	–1.2055	0.005518
TG 60:13|TG 18:2_20:5_22:6	0.47613	–1.0706	0.024815
TG 62:15|TG 18:3_22:6_22:6	0.49383	–1.0179	0.013163
TG 66:18|TG 22:6_22:6_22:6	0.46664	–1.0996	0.015577

a Threshold: *p* < 0.05 and FC > 2. Fold change (FC), log (base 2) fold change (log2.FC), unadjusted *p* value (raw.pval).

## 4 Discussion

Previous transcriptomics studies have predicted altered lipid synthesis and metabolism in developing fish exposed to crude oil or phenanthrene alone.^8,15−17^ It has been hypothesized that phenanthrene or crude oil exposure may alter the embryonic lipid profile by impairing heart function, thus reducing the embryo’s ability to mobilize nutrients from the maternally deposited yolk.^16,19^ Contrary to this hypothesis, we demonstrate that total lipids are significantly reduced in the yolk of zebrafish embryos exposed to phenanthrene (Figure 3B). The current study used the same doses and exposure regime as McGruer et al. (2021),^19^ which found an increase in yolk area at 12 μM phenanthrene. This result suggests an increased demand for, and ability to mobilize, lipid resources with phenanthrene exposure despite cardiac impairment. Further supporting this, there were no metabolite classes where relative abundance was increased in the yolk while being reduced in the embryo body (Figure 5). However, the yolk contains both proteins and lipids. Because we did not evaluate protein content we cannot rule out that the increase in yolk size could be due to underutilization of proteins or impaired osmoregulation resulting in fluid accumulation and reduced density of yolk nutrients. Thus, measurement of two-dimensional yolk area can provide insight into yolk usage but needs validation.

Investigating the yolk composition revealed several classes of lipids that were altered following phenanthrene exposure. TGs are an essential energy storage molecule in fish.^32^ Previous work by Laurel et al. (2019),^18^ investigating the impacts of crude oil in polar cod, found significant perturbation to TGs with exposure to crude oil. In cod, TGs were not significantly impacted during the 7-day exposure and washout period.^18^ However, TGs were increased during the larval growth period and significantly reduced at time points following absorption of the yolk. In the current study, we also found TG disruption in zebrafish with exposure to phenanthrene (Figure 5). Yet, the stage at which this occurs and the direction of the TG changes in our study differed from polar cod. In zebrafish, we found an immediate reduction in TGs in both the yolk and embryo body following exposure when the embryos still have a substantial yolk sac (72 hpf). Oil Red O staining for neutral lipids was also reduced in the embryo body with exposure at this stage, further supporting this result (Figure 2). While we can conclude that both oil exposure and phenanthrene disrupt neutral lipids, the species, life history, and severity of exposure likely play an important role. Polar cod live in a cold environment and need approximately 50 days until hatch,^18^ not fully utilizing their yolk until about 3 weeks later (∼70 dpf).^33^ Zebrafish embryos develop quickly, hatching within 2–3 days of fertilization and completely using their yolk by 5 dpf.^20^ Yolk composition also varies by species,^34^ and lipid composition in fish can depend on water temperature.^35^ Mahi mahi embryos, a warm water species, exhibited an increased energy demand and reduced yolk area with elevated temperature, oil exposure, UV exposure, or a combination of these stressors.^36^ Changes to lipid reserves as embryos transition from a yolk nutritional supply to free feeding have been shown to influence larval survival.^37^

Looking at changes to distinct classes of lipids, we can begin to piece together the broad patterns of how lipids are altered following phenanthrene exposure. DGs are intermediates in the anabolism and catabolism of TG, PCs, and PEs.^38^ Total DGs were significantly reduced with phenanthrene exposure (Figure 5). This reduction could be due to an elevated demand for downstream molecules, such as TGs, which were significantly reduced with exposure. DGs are also important signaling molecules and regulators of ion channel function and transporters.^39,40^ Furthermore, DGs can be converted to PCs and PEs, which collectively account for over 50% of eukaryotic membrane phospholipids.

In the 72 hpf embryo body, we see divergent changes to PEs and PCs. PEs were significantly reduced in both the embryo body and yolk, while PCs were significantly increased in the embryo body (Figure 5). Changes to these phospholipids individually, and disruption of the PC/PE ratio, have been implicated in numerous diseases. An increase in the PC/PE ratio in the mitochondrial membrane impairs mitochondrial function, which may impact ATP production.^41^ Mitochondrial impairment was predicted to be altered in crude oil exposed red drum embryos.^17^ Additionally, an elevated PC/PE ratio has been associated with decreased SERCA-mediated calcium uptake resulting in muscle weakness in mouse skeletal muscle.^41^ Fish cardiomyocytes exposed to phenanthrene or crude oil, display reduced Ca^2+^ flux through SERCA.^12,13^ Though this change to cardiomyocyte function occurred rapidly after exposure and may not solely be due to changes in membrane composition, changes in lipid composition could exacerbate these effects.

Pathway analysis highlighted changes to the lysophospholipids LPC and LPE. Lysophospolipids can be supplied by hydrolysis of membrane phospholipids.^42^ LPC can be produced from PC, likewise LPE from PE. Ontology analysis revealed significant increases to LPE in the 72 hpf body and increases to LPC in both the embryo body and yolk (Figure 5). Using a more stringent threshold (*p* < 0.05 and fold change >2) for metabolites in 72 hpf body samples, we found that many of the identified LPCs and LPE were some of the most significantly impacted individual lipids (Table 2). Furthermore, pathway analysis highlighted similar findings, predicting that increases in several LPE and LPCs could cause the development of a disease model associated with inflammation and neuronal demyelination (Figure 6). LPC is associated with demyelination, leading to motor function defects,^43^ and has long been used in an experimental setting to induce demyelination.^44^ Recently, work exploring the mechanism of LPC demyelination demonstrated that LPC can integrate into the cell membrane, which induces permeability and eventually cell death.^44^ Likewise, LPE is elevated in the sera of patients with multiple sclerosis and the brains of Alzheimer’s disease (AD) mouse models, both neurological diseases (reviewed by Leuti et al. 2020).^40^ Recovery in an AD model was associated with lower LPE levels.^45^ Exposure to crude oil or phenanthrene alters behavioral responses, which may be caused by damage to the central and peripheral nervous systems.^11^ The transcriptome of red drum larvae exposed to crude oil indicated inhibited neuron development, and phenotypically larvae developed smaller brains.^17^ However, the mechanisms underlying these responses have yet to be identified. Neurological effects from elevated LPE and LPCs are one possible mechanism. LPC can also induce inflammation and modulate immune function by activating specific receptors and signaling pathways.^46^

Mechanisms underlying the phenanthrene-induced increase in LPC and LPE warrant further study. Ingenuity Pathway Analysis predicted the production of several LPCs by Sirtuin 6 (SIRT6) and a relationship between LPC 18:1 and patatin-like phospholipase 6 and 7 (PNPLA6 and PNPLA7) (Figure 6). SIRT6 is a chromatin-associated NAD^+^-dependent deacetylase^47^ that can lead to transcription of a large number of target genes in response to stress-signaling.^48^ Knockout of SIRT6 is associated with increased transcription of genes in the phospholipase A2 (PLA_2_) family,^49^ which can lead to the production of lysophospholipids.^50^ Several phospholipase A2 enzymes were predicted to be altered in studies assessing oil exposure on 96 hpf mahi mahi embryos^15^ and 72 hpf red drum embryos.^17^ PNPLA6 and PNPLA7 have also been shown to have direct LPC hydrolase activity.^51,52^

## 5 Conclusion

Overall, we demonstrate the benefits of removing, or separately profiling, the embryonic yolk sac to refine result interpretation in lipidomics studies. By profiling the yolk, we found that at phenanthrene concentrations that caused cardiotoxicity, total lipid metabolites were significantly reduced within the yolk. This reduction demonstrates that an increase or decrease in two-dimensional yolk area does not alone provide enough evidence to indicate disruption of yolk nutrients and suggests that lipid impacts are not caused by reduced yolk utilization due to cardiac disruption. Similarly, TG and neutral lipids were significantly reduced in the 72 hpf embryo body and TG was also reduced in the yolk, suggesting an increase in energy usage in exposed embryos. Finally, nontargeted lipidomics allowed us to take an unbiased look at lipids altered by exposure, highlighting LPC and LPE species increase with phenanthrene exposure. Changes in LPC and LPE concentrations have been linked to disease outcomes in many species and could contribute to neurotoxicity.

Our study is limited in that it could only capture effects at one dose and profile the yolk at one life stage. It would be interesting to see how the lipid profile changes across doses, especially at doses that do not induce visible cardiotoxicity. Consistent with other lipidomics studies, we could only identify a fraction of the thousands of lipids in our samples. Our data will be available in the Dryad data repository (10.6086/D1ZH5T). As metabolomic libraries improve, we hope these data can be revisited.

## Data Availability

Data used in this study is available in the Dryad data repository (10.6086/D1ZH5T).

## Supporting Information Available

Supplemental figures: survival, metabolite heatmap, pathway analysis (PDF)
