# Supplementary material for: Lipid Profile Altered in Phenanthrene Exposed Zebrafish Embryos with Implications for Neurological Development and Early Life Nutritional Status
Source: Environ Health (Wash). 2023 May 30;1(1):32–40. doi: 10.1021/envhealth.3c00002 (PMC11504619; doi:10.1021/envhealth.3c00002)
Supplement: Supplementary file 1 — eh3c00002_si_001.pdf [file eh3c00002_si_001.pdf]

Lipid profile altered in phenanthrene exposed zebrafish embryos with implications for neurological development and early life nutritional status

Victoria McGruer[1*], Anil Bhatia[2], Jason T. Magnuson[3] Daniel Schlenk[1]

[1]Department of Environmental Sciences, University of California, Riverside, CA, USA

[2]Metabolomics Core Facility, IIGB, University of California, Riverside, CA, USA

[3]Department of Chemistry, Bioscience and Environmental Engineering, University of Stavanger, Stavanger, Norway

*Corresponding author:

Victoria McGruer

Victoria.McGruer@ucr.edu

Department of Environmental Sciences, University of California Riverside, 2460A Geology, Riverside, CA 92521, United States

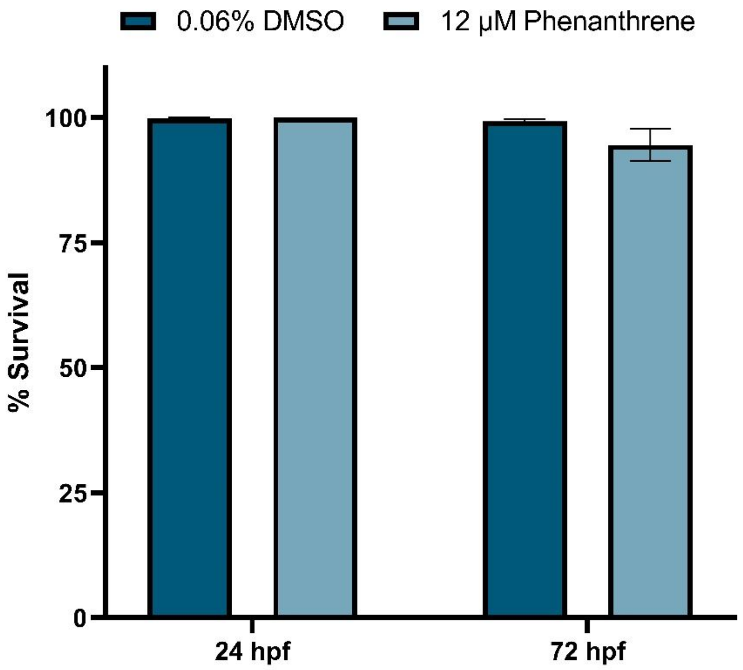

Figure S1 – Percent embryo survival following exposure to 0.06% DMSO or 12 µM Phenanthrene prior to sampling at 24 or 72 hpf. Error bars represent mean ± SEM.

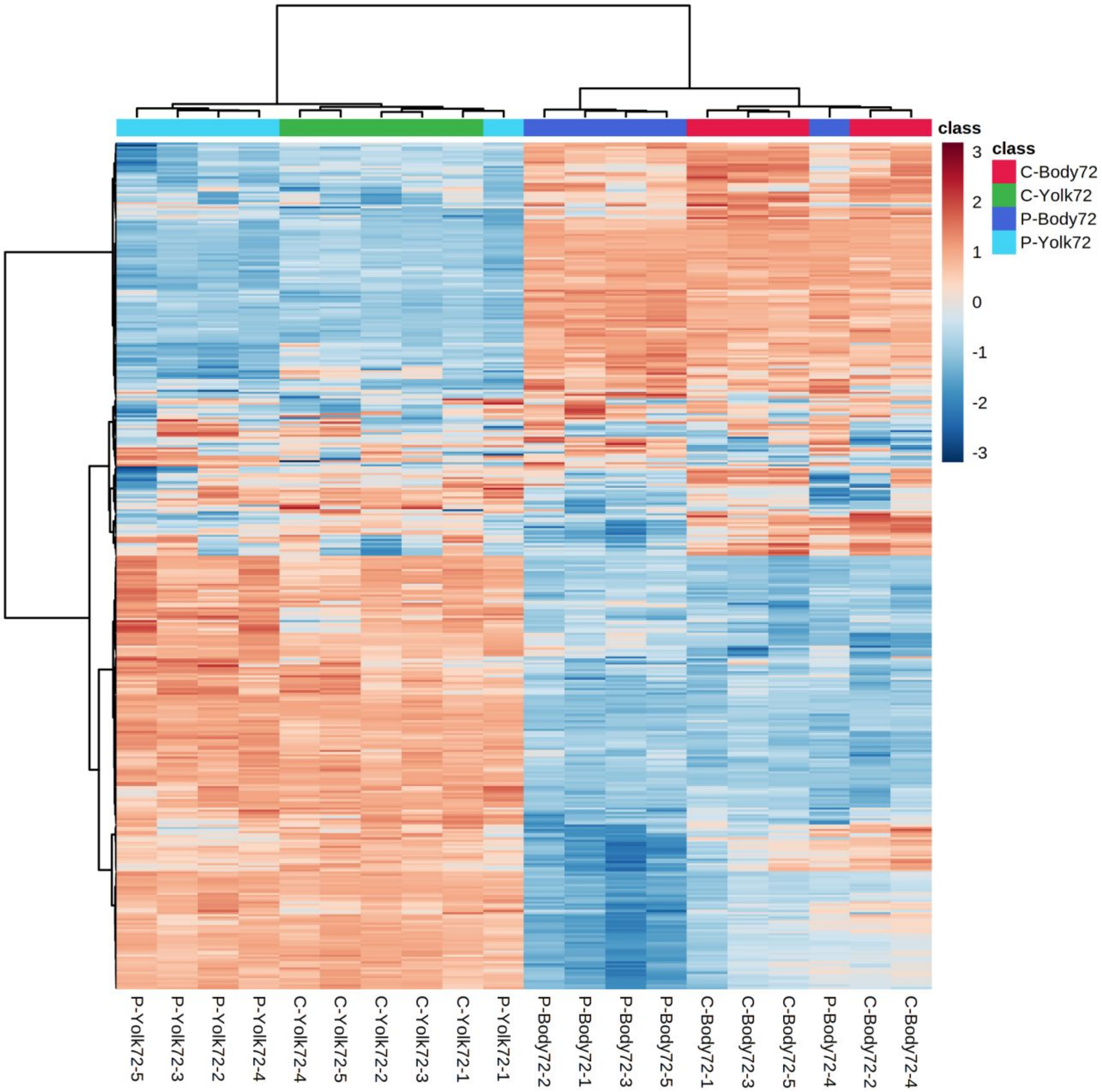

Figure S2 – Relative abundance of metabolites in "body" or "yolk" samples collected from 72 hpf zebrafish embryos exposed to 0.06% DMSO or 12 µM Phenanthrene. Values are autoscaled (mean-centered and divided by the standard deviation of each variable). Each row represents an individual metabolite. Each column is a biological replicate.

Diseases and Disorders

Name	p-value range	# Molecules
Immunological Disease	2.94E-10 - 2.94E-10	5
Inflammatory Disease	7.10E-07 - 2.94E-10	6
Inflammatory Response	1.12E-02 - 2.94E-10	7
Neurological Disease	2.94E-10 - 2.94E-10	5
Organismal Injury and Abnormalities	1.12E-02 - 2.94E-10	7

Table S1- Top diseases and disorders predicted by Ingenuity Pathway Analysis based on lipids that were significantly altered ($p < 0.05$) in 72 hpf embryo body samples following phenanthrene exposure.
